# Incidental mosquitocidal effect of an ivermectin mass drug administration on *Anopheles farauti* conducted for scabies control in the Solomon Islands

**DOI:** 10.1093/trstmh/trx025

**Published:** 2017-06-13

**Authors:** Christian Kositz, Jeptah Talina, Jason Diau, Rowena Asugeni, Cheryl Whitehorn, David Mabey, Carlos Chaccour, Michael Marks

**Affiliations:** a Clinical Research Department, Faculty of Infectious and Tropical Diseases, London School of Hygiene & Tropical Medicine, London, UK; b Atoifi Adventist Hospital, Atoifi, Malaita, Solomon Islands; c Faculty of Infectious and Tropical Diseases, London School of Hygiene & Tropical Medicine, London, UK; d Hospital for Tropical Diseases, University College London Hospitals NHS Trust, London, UK; e ISGlobal, Barcelona Centre for International Health Research (CRESIB), Hospital Clínic - Universitat de Barcelona, Barcelona, Spain

**Keywords:** *Anopheles farauti*, Ivermectin, Malaria, Solomon Islands

## Abstract

**Background:**

The Solomon Islands is targeting elimination of malaria by 2030. The dominant vector is the predominantly exophagic, exophilic *Anopheles farauti sensu strictu*. This biting behaviour limits the efficacy of conventional vector control tools and highlights the need for new strategies. When administered to humans ivermectin has been shown to have a mosquitocidal effect. Mass drug administration (MDA) with ivermectin is an emerging strategy in the control of scabies. In this study we explored any incidental effect of ivermectin MDA conducted for scabies control on mosquitoes.

**Methods:**

MDA for scabies was conducted in three villages. We performed human landing catches and measured 5-day mortality amongst *Anopheles* mosquitoes caught before and after MDA. Cox regression was used to calculate hazard ratios (HR) for mortality between mosquitoes caught before and after MDA.

**Results:**

There was a significant increase in 5-day mortality in anopheline mosquitoes caught post-MDA which was highest on the day of MDA itself (HR 4.2 95% CI 1.8 to 10.1, p=0.001) and the following day (HR 4.4 95% CI 1.8 to 10.8, p=0.002) compared to mosquitoes caught before MDA.

**Conclusions:**

This study shows a possible mosquitocidal effect of ivermectin MDA conducted for scabies control. Studies with a larger sample size with clinical as well as entomological outcomes should be conducted in this population.

## Introduction

WHO has set an ambitious goal to eliminate malaria from 35 countries by the year 2030.^1^ The Solomon Islands is one of the countries targeted by WHO for malaria elimination. The country is an archipelago, consisting of approximately 1000 islands, with a population of approximately 500 000.^2^ In 2014 the rate of confirmed cases of malaria was 44 per 1000 person years.^3^ Malaria has been reported to have declined as a cause of fever in some parts of the Solomon Islands in the last ten years^4^ but remains an important public health problem.

Prior to the Malaria Eradication Programme (MEP) in the 1970s three species of *Anopheles* mosquitoes were involved in the transmission of malaria in the Solomon Islands. *Anopheles koliensis, Anopheles punctulatus* and *Anopheles farauti sensu strictu*. The MEP was based predominantly on a strategy of indoor residual spraying (IRS) combined with mass drug administration.^5^*An. koliensis* appears to have been eliminated following the MEP and *An. punctulatus* is no longer considered a major vector having been controlled through the use of IRS and long-lasting insecticide treated bed nets (LLINs) in the 1990s.^6^*An. farauti s.s.* is now considered to be the dominant vector for malaria in the Solomon Islands.^7^ Compared *to An. gambiae, An. farauti* is a relatively ineffective vector, with a relatively short life span and indiscriminate host preference, which results in highly variable rates of human biting.^8–10^

The selective pressure exerted by the MEP resulted in a new behavioural pattern of *An. farauti s.s.* This species now feeds and rests predominantly outdoors and has peak biting activity outside sleeping hours,^11,12^ which reduces the efficacy of IRS and LLINs^13^ as methods of vector control. New vector control tools might be required to achieve the 2030 elimination target.

There is increasing interest in the potential role of ivermectin as a complementary tool to reduce malaria transmission.^14,15^ The drug reduces the survival of insectary-bred anophelines that feed on volunteers, and those caught in the wild including *An. farauti*.^15,16^ This effect is mediated by antagonism of mosquito glutamate-gated chloride channels resulting in flaccid paralysis and death.^14^ A small number of studies have also assessed the incidental effect of ivermectin mass drug administration (MDA) conducted for both lymphatic filariasis and onchocerciasis on local mosquito populations. These studies have shown increased mortality amongst African malaria vectors^17–19^ but few similar field studies have been conducted in the Pacific region^20^ and none directly assessing *An. farauti s.s*.

Ivermectin is also emerging as a key tool in the control of scabies. MDA with ivermectin has been shown to have a dramatic effect on the prevalence of scabies.^21^ The Pacific region is a particular focus for scabies worldwide^22–24^ and a number of large scale evaluations of ivermectin MDA are either underway or planned in the region. Given the biting habits of *An. farauti s.s.*, ivermectin MDA might be of use in the region as a complementary vector control tool.

Given the high prevalence of scabies in the Solomon Islands and the 2030 malaria elimination target there are important potential synergies between the NTD and malaria control programmes. This study was conducted to assess whether ivermectin MDA conducted for the purposes of scabies control could have an incidental mosquitocidal effect on *An. farauti s.s.* and other local malaria vectors.

## Methods

### Study site

The study was conducted in communities in the East Kwaio region of the Solomon Islands. Three communities located in Uru Harbour were selected to participate in this study due to their involvement in the scabies intervention study that was being conducted in these communities. The three communities were all located within 750 m of each other and were considered as a single site for the purpose of this study. All villages were located in a coastal region where *An. farauti s.s*. is known to be the dominant anopheline mosquito.

**Figure 1.**
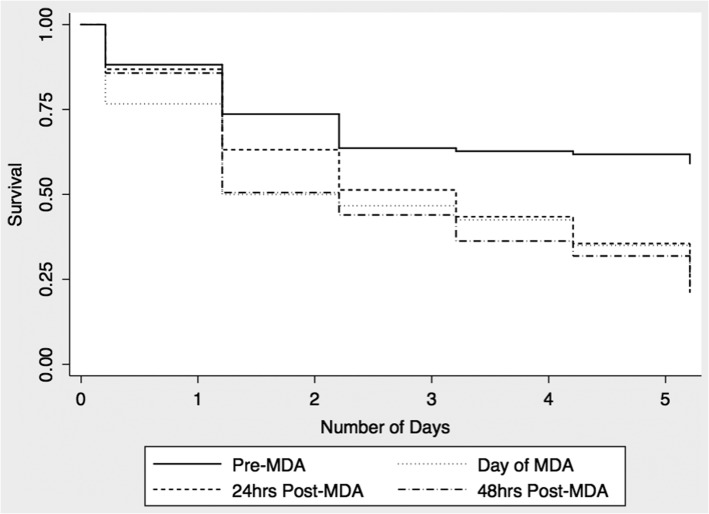
5-day survival of anopheline mosquitoes.

**Figure 2.**
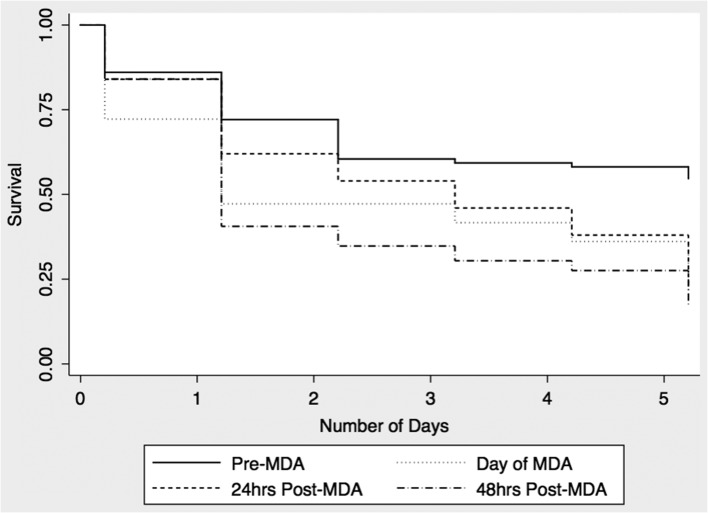
5-day survival of *Culex* mosquitoes.

### Mass drug administration

This entomological survey ran alongside a scabies intervention study [ClinicalTrials.gov identifier: NCT02775617]. Briefly, MDA with single dose ivermectin (200 μg/kg) was conducted between the hours of 10:00 h and 18:00 h as previously described.^21^ All individuals in the village were invited to participate, including the human landing catchers (HLC). Pregnant and breastfeeding women and children weighing less than 15 kg were excluded from ivermectin mass treatment in line with treatment guidelines. The MDA achieved 80% coverage. For the purpose of this study, the day that MDA was conducted was considered as day 1 for ivermectin exposure.

### Field work

Adult volunteers (aged>18 years) to perform the human landing catches were enrolled from the local communities. Volunteers were split into two groups (four pairs and one group of three). A member of the study team (CK) trained volunteers to perform human landing catches as previously described^25–27^ and in line with WHO 2013 guidelines for entomological and vector control participants.^28^ Catches were supervised by a member of the study team (CK). Briefly each individual was provided with a mouth aspirator, head torch and a net covered cup for collecting mosquitoes. Each of the catchers received their dose of ivermectin at the same time, around 16:00 h. Volunteers were spread out across the communities and performed catches outdoors under an open wooden roof in front of the houses. To avoid bias the same sites were used for human landing catches on each day of the study. In line with the known biting habits of *An. farauti s.s.* from other sites. Catching was performed between 18:00 h−23:00 h.^12^ Volunteers were checked for fever routinely during the study period and for a two-week observation following the final landing catch. At the end of the observation period all individuals were offered a rapid diagnostic test for malaria (CareStart Malaria, Access Bio, Somerset, New Jersey, USA). Individuals who had a positive rapid diagnostic test were provided with a full treatment course of arthemeter-lumefantrine free of charge.

All mosquitoes caught were stored in a central room with screened open windows to guarantee equal humidity and temperature between the room and the environment, and nutrition was maintained with cotton wool pads impregnated with a 10% sugar solution that was exchanged daily. Partially engorged and unfed mosquitoes were not discarded and were included in the analysis.

Collections took place for 4 consecutive days (1 day before MDA and the 3 subsequent days). This duration was selected based on the half-life of ivermectin as the plasma level falls below the level which is felt to be mosquitocidal after 3 days. Each collection was reviewed daily at 23:30 h. Dead mosquitoes were separated from live mosquitoes. Identification of the dead mosquitoes was performed the subsequent day. Identification was based on direct visual inspection and magnified inspection using an Olympus X31 microscope (Olympus, Tokyo, Japan) at X40 magnification. Mosquitoes were classified as *Anopheles* or *Culex*. Although there are other *Anopheles* species that bite humans in the Solomon Islands, *An. farauti s.s.* is the only coastal dwelling and brackwater breeding anopheline mosquito biting humans in the Solomon Islands. The other species do not share this specific biological niche with *An. farauti s.s.*^10^ therefore, it was expected that the anophelines caught would be *An. farauti s.s.*,^29^ but PCR for speciation was not performed.

### Statistics

The primary endpoint of the study was the 5-day mortality of *Anopheles* species mosquitoes. For the purposes of analysis, we pooled mosquitoes caught across the three communities each day. Based on previous data on the effect of ivermectin on mosquitoes we aimed to compare mortality in mosquitoes caught before MDA and up to 3 days after MDA. Based on existing data the predicted 5-day mortality of mosquitoes caught pre-MDA was expected to be 20% while that of mosquitoes caught on the first days after MDA was expected to be close to 70%.^18,30^ Based on these data we calculated that at least 19 anopheline mosquitoes were required per night across all HLCs combined in order to have 90% power to detect this difference. Kaplan-Maier curves were constructed and Cox regression was used to calculate hazard ratios for 5-day mortality for mosquitoes caught before ivermectin MDA and on the 3days following MDA. The analysis was performed in STATA 13.1 (Statacorp, College Station, Texas, USA).

### Study ethics

Individuals performing human landing catches provided informed written consent to participate in the study. HLC volunteers did not receive prophylaxis, as studies have shown that they are not at a higher risk of acquiring malaria compared to the general population.^25^

## Results

A total of eleven male volunteers (median age 38, IQR 30–44) were enrolled in the study to perform the landing catches. Ten of the individuals were asymptomatic throughout the study period and the subsequent 2-week observation period. All had a negative malaria rapid diagnostic test. One individual was asymptomatic but his rapid diagnostic test was positive for *Plasmodium falciparum* HRP2 and he was treated with a full course of arthemeter-lumefantrine, and was well at 1 week follow-up.

A total of 110 mosquitoes were caught pre-MDA across the three communities of which 24 (21.8%) were anopheline. On the day of MDA 120 mosquitoes were caught of which 48 (40.0%) were anopheline. Seventy-six mosquitoes were caught the following day of which 26 were anopheline (34.2%). On the final day of catching 97 mosquitoes were caught of which 22 (22.6%) were anopheline (Table 1).

**Table 1.** Species of mosquitoes caught by day

Species	Pre-MDA	Day of MDA	24 h post-MDA	48 h post-MDA	Total
*Anopheles*	24 (21.8%)	48 (40.0%)	26 (34.2%)	22 (22.6%)	120 (30.2%)
*Culex*	86 (78.1%)	72 (60.0%)	50 (65.8%)	69 (77.4%)	277 (69.8%)
Total	110	120	76	91	397

MDA: Mass drug administration.

The 5-day mortality for pre-MDA anopheline mosquitoes was 25%. The respective 5-day mortalities for anopheline mosquitoes caught post-MDA were 75% on day 1, 80% on day 2 and 68% on day 3 (Figure 1). Mortality was significantly increased for mosquitoes caught each of the 3 days post MDA (Table 2). Mortality was highest in mosquitoes caught on day 1 (HR 4.2, 95% CI 1.8 to 10.1) and day 2 (HR 4.4, 95% CI 1.8 to 10.8). We also noted increased 5-day mortality of *Culex* mosquitoes although the effect size was smaller than that noted for *Anopheles* species (Figure 2 and Table 2) No *Aedes* mosquitoes were caught during the experiments.

**Table 2.** Hazard ratios for 5-day mortality

Mosquito species	Day of capture^a^	5-day mortality HR (95% CI)^b^	p-value^c^
*Anopheles*	1	4.2 (1.8 to 10.1)	0.001
	2	4.4 (1.8 to 10.8)	0.002
	3	3.1 (1.2 to 8.1)	0.018
*Culex*	1	1.9 (1.3 to 2.9)	0.002
	2	1.9 (1.2 to 3.0)	0.004
	3	2.3 (1.5 to 3.5)	<0.001

HR: Hazards ratio; MDA: Mass drug administration.

^a^ Day of capture in relation to MDA. Day 1 indicates mosquitoes were caught the evening following MDA.

^b^ Hazard ratios compared to mosquitoes caught pre-MDA.

^c^ Wald Test.

## Discussion

In this field study we have seen an incidental but significant increase in mortality of *Anopheles* caught up to 3 days after ivermectin MDA for scabies control. As ivermectin MDA may be scaled up for the control of scabies in the Pacific region the incidental impact on mosquitoes is worthy of further study. This study adds to existing data^16,20^ and suggests that a single dose of ivermectin MDA for scabies control could have a possible impact on the survival rate of *Anopheles* in the Solomon Islands, where the main vector can elude control with LLINs and IRS, and biting rates are comparatively low. While an effect of ivermectin on *An. farauti s.s.* has previously been documented in volunteer-feeding studies, the current study is to our knowledge the first field study assessing the mortality of *An. farauti s.s.* after ivermectin MDA in this area.

Mosquito mortality was higher in those caught within the first 48 hours of MDA and had begun to decline by day 3. This trend is in line with the known pharmacokinetics of ivermectin as reduced plasma levels are expected 3 days after a single 200 μg/kg dose.^31,32^ It has to be noted though that the mortality rates seen on day 1 and day 2 could have purely been caused by mosquitoes biting the HLCs only. However, village life did continue during catches and therefore, the collected mosquitoes may have represented a mixture of those who feed on catchers and villagers, with some of the mosquitoes biting villagers as well as catchers to complete a full blood meal.

The main limitations of this study are the small number of mosquitoes caught, the limited time frame in which the study was conducted and the lack of a parallel control site. It was not possible to do parity dissections to account for previous blood meals and age distribution of mosquitoes which might have affected our findings, as we could not determine the age structure of our mosquitoes. However, a study conducted in Northern Guadacanal showed only a seasonal change in density not in age structure.^11^ This study was conducted at a time when mosquito density has previously been reported to on the lower end for *Anopheles* mosquitoes.^10,13^ Despite this the number of anopheline mosquitoes caught was in line with our target sample size. Although mosquitoes were caught for only a limited number of hours each day it has been previously documented that the peak biting period occurs between 18:00 h–21:00 h, which is the time period chosen for mosquito catches in this study.^12^

Modeling studies suggest that large scale ivermectin MDA with an effect size similar to that seen in this study would be anticipated to result in reductions in malaria transmission.^33^ Larger field studies are needed to confirm this and consideration should be given to assessing both entomological and clinical malaria endpoints in other scabies MDA studies in the region. If confirmed in larger studies this might represent an opportunity for integration and synergies between vector borne disease and NTD control programmes.

*An. farauti s.s.* has indiscriminate host feeding preferences and will also readily feed on livestock such as pigs. Treatment of pigs with ivermectin may also be a viable strategy in this setting where communities live in close proximity to livestock. In this setting, where vectorial capacity is already relatively low, the addition of ivermectin MDA solely as a complementary vector control tool to the already existing ones may facilitate interruption of transmission and ultimately elimination of malaria in the Solomon Islands. It would be helpful to assess this possibility and the required number of ivermectin treatments through future modelling using the available entomological data.

The effect seen on the 5-day mortality rate in *Culex* mosquitoes warrants further research, as culicine mosquitoes have been reported to be less susceptible to ivermectin.^34,35^ As culicines were also not identified in more detail, it was not possible to identify specific species and their susceptibility to ivermectin.
